# Development and application of a competitive ELISA for the detection of antibodies against *Salmonella* Abortusequi in equids

**DOI:** 10.1128/jcm.00273-23

**Published:** 2023-10-24

**Authors:** Kui Guo, Wei Guo, Diqiu Liu, Weiguo Zhang, Yan Yang, Zenan Zhang, Shuaijie Li, Jinhui Wang, Xiaoyu Chu, Yaoxin Wang, Zhe Hu, Xiaojun Wang

**Affiliations:** 1 State Key Laboratory for Animal Disease Control and Prevention, Harbin Veterinary Research Institute, the Chinese Academy of Agricultural Sciences, Harbin, China; Boston Children's Hospital, Boston, Massachusetts, USA

**Keywords:** *S*. Abortusequi, cELISA, antibody, equine, *Salmonella*, abortion

## Abstract

The high abortion rate associated with *Salmonella* Abortusequi (*S*. Abortusequi) infection in equids has re-emerged over the past 10 years and has caused serious economic losses to China. Our previous studies showed that the flagellin FljB gene could distinguish *S*. Abortusequi from most *Salmonella* serotypes. In this study, the flagellin antigen was used to develop a competitive enzyme-linked immunosorbent assay (cELISA) that could be used to detect both horse and donkey serum samples using a monoclonal antibody (MAb) that was found to bind to FljB. A cELISA was established using the purified MAb coating of the plate and incubation of the mixture of horseradish peroxidase (HRP)-conjugated FljB antigen with the undiluted serum sample. The performance of the cELISA and the tube agglutination test (TAT) assay was compared with respect to sensitivity and specificity, by testing a panel containing 660 *S*. Abortusequi-positive and 515 *S*. Abortusequi-negative serum samples, all of which had been characterized by Western blotting. Receiver operator characteristic (ROC) analyses were performed to determine the cutoff value and estimate the detection specificity (Sp) and sensitivity (Se). ROC analysis showed that the area under the ROC curve (AUC) values of cELISA [AUC = 0.9941; 95% confidence interval (CI), 0.9898–0.9984] were higher than those of TAT (AUC = 0.7705; 95% Cl, 0.7437–0.7972). A cutoff value of 39.5% was selected with Sp and Se values of 100 (95% Cl, 99.26–100.00) and 97.58 (95% Cl, 96.10–98.50), respectively. The cELISA has excellent futures compared with TAT, such as shortened detection time, no need for pre-treatment of sera, and easy interpretation of the results, and is more suitable for disease surveillance.

## INTRODUCTION

*Salmonella* represents one of the most common Gram-negative bacterial pathogens and causes a variety of symptoms including gastroenteritis, food poisoning, typhoid fever, diarrhea, bacteremia, and others (1, 2). *Salmonella* is categorized into more than 2,600 *Salmonella* serotypes (3), mainly depending on lipopolysaccharide (O antigen) and flagellin (H antigen) (2, 4 – 9). *Salmonella enterica* subsp. *enterica* serovar Abortusequi (*S*. Abortusequi) is one of the important pathogens causing an abortion in mares, as well as associated with neonatal septicemia, multiple abscesses, orchitis, and polyarthritis in equid hosts (10). Furthermore, *Salmonella* Typhimurium (*S*. Typhimurium), *Salmonella* Enteritidis (*S*. Enteritidis), and *Salmonella* Dublin (*S*. Dublin) may also cause this disease in equids in some cases (11).

*S*. Abortusequi was first described in 1893 in the USA and has subsequently been found in both horses and donkeys from multiple geographic areas across Italy (12, 13), Croatia (14, 15), Argentina (16, 17), Russia (18), India (19, 20), Australia (21), and Japan (10, 22, 23). In China, the high abortion rate associated with *S*. Abortusequi infection in equids has re-emerged over the last decade, and currently, the abortion rate in infected mares is between 30% and 100% (24). The significant economic losses in equine husbandry in China are mainly due to the growing number of abortions caused by *S*. Abortusequi infection (25). Moreover, etiological data based on bacteria isolation have provided evidence of the widespread circulation of *S*. Abortusequi among horse and donkey farms in Inner Mongolia, Xinjiang, Shandong, Heilongjiang, and Hebei provinces in China (24 – 26).

The gold standard for detecting carriers of *S*. Abortusequi is the detection of the live agent, but this has the disadvantage of being both time-consuming and laborious (27). In addition, vaginal swab samples, fetal tissues, or fecal detection can result in false negatives even in farms at which an outbreak of *S*. Abortusequi infection has occurred (28). In this case, serological assay detection is considered to be a good complementary method to detect *S*. Abortusequi infections. The serological assay has excellent futures compared with bacteria isolation, such as shortened detection time, and can be used for monitoring asymptomatic animals. Furthermore, it can be used as a tool for large-scale serological surveys for the detection of antibody against *S*. Abortusequi. Currently, the most common method for measuring serum antibody against *S*. Abortusequi is the tube agglutination test (TAT) (15, 23). However, the World Organization for Animal Health (WOAH) reports that the TAT is not highly specific and may cross-react with antibodies from other Enterobacterales (29).

Another option for the detection antibodies against *S*. Abortusequi is the enzyme-linked immunosorbent assay (ELISA) assay and includes both indirect (iELISA) and competitive (cELISA) ELISAs. The main advantage of cELISAs over iELISAs is that the cELISAs for the detection of antibodies enable the testing of samples from different animal species. To the best of our knowledge, no specific cELISA serological method capable of the specific identification of *S*. Abortusequi has been published to date. Previous studies have identified flagellin as a potential candidate for the serological detection of *Salmonella*, as the protein is abundantly expressed, located on the surface of the bacteria, and has good antigenicity (1, 30 – 33). Previous research has resulted in an iELISA based on recombinant flagellin for the detection of antibodies against *S*. Enteritidis and has demonstrated that the assay has good specificity and did not cross-react with other bacteria (1). Our previous studies showed that the flagellin FljB in nucleotide or amino acid could distinguish *S*. Abortusequi from most *Salmonella* serotypes (24, 34). In this study, a cELISA based on a monoclonal antibody (MAb) against flagellin FljB, for the detection of *S*. Abortusequi antibodies in the sera of different animal species was developed and evaluated.

## MATERIALS AND METHODS

### Serum samples

A total of 1,175 sera including 660 *S*. Abortusequi positive and 515 S. Abortusequi negative serum samples were used in this study (Table 1). One standard positive serum was collected from a horse naturally infected with *S*. Abortusequi in Heilongjiang for the development of the cELISA. One positive serum from a rabbit experimentally immunized with *S*. Abortusequi was purchased from the China Institute of Veterinary Drugs Control. The other positive serum samples were collected from farms where *S*. Abortusequi infection is known to have occurred in Inner Mongolia, Xinjiang, Shandong, and Hebei provinces in China, during 2018–2022. Most of the samples were collected from horses and donkeys in the farms with a clinical abortion caused by *S*. Abortusequi infection, which were confirmed with bacteria isolation and Western blotting. Ten antisera positive against equine herpes virus (EHV), equine influenza virus (EIV), equine arteritis virus (EAV), equine infectious anemia virus (EIAV), *Theileria equi*, *Babesia caballi*, *Streptococcus equi*, *S*. Typhimurium, *S*. Dublin, and *S*. Enteritidis were selected for the analytical specificity test. Antisera against EHV, EIV, or EAV were purchased from National Veterinary Services Laboratories Inc in the USA. Antisera against EIAV, *S. equi*, *T. equi*, *B. caballi*, *S*. Typhimurium, *S*. Dublin, or *S*. Enteritidis were prepared using healthy horses and stored in our laboratory, which were tested specific to the mentioned pathogens using commercial antibody detection kits. Negative serum samples were collected from healthy horses or donkeys in Xinjiang, Chongqing, Gansu, Henan, Jiangsu, and Hubei provinces.

**TABLE 1:** Description of serum samples used in this study

Serum classification	No. of sera	Species	Western blot results	cELISA^*a*^	TAT^*b*^	TAT (titers)	Anamnestic data
Positive total = 660	1	Horse	Pos	Pos	Pos	64	From a horse naturally infected with *S*. Abortusequi in Heilongjiang.
	1	Rabbit	Pos	Pos	Pos	4	From a rabbit experimentally immunized with *S*. Abortusequi.
	103	Horse	Pos	Pos	Pos	1–32	Serum samples were collected from farms where *S*. Abortusequi infection is known to have occurred in Inner Mongolia, Xinjiang, Shandong, and Hebei provinces in China, during 2018–2022. Most of the samples were collected from horses and donkeys in the farms with a clinical abortion caused by *S*. Abortusequi infection, which were confirmed with Western blotting.
	252	Donkey	Pos	Pos	Pos	1–32	
	140	Horse	Pos	Pos	Neg	<1	
	150	Donkey	Pos	Pos	Neg	<1	
	6	Horse	Pos	Neg	Neg	<1	
	7	Donkey	Pos	Neg	Neg	<1	
Negative total = 515	3	Horse	Neg	Neg	Neg	<1	Antisera against EHV,^*c*^ EIV,^*d*^ and EAV^*e*^ were purchased from National Veterinary Services Laboratories, Inc in the USA.
	7	Horse	Neg	Neg	Neg	<1	Antisera against EIAV,^*f*^ *S. equi*, *S*. Dublin, *S*. Typhimurium, *S*. Enteritidis*, T. equi*, and *B. caballi* were prepared using healthy horses.
	339	Horse	Neg	Neg	Neg	<1	Collected from healthy horses in Xinjiang, Chongqing, and Gansu.
	166	Donkey	Neg	Neg	Neg	<1	Collected from healthy donkeys in Henan, Jiangsu, and Hubei.

^*a*^ cELISA, competitive enzyme-linked immunosorbent assay.

^*b*^ TAT, agglutination test.

^*c*^ EHV, equine herpes virus.

^*d*^ EIV, equine influenza virus.

^*e*^ EAV, equine arteritis virus.

^*f*^ EIAV, equine infectious anemia virus.

### Bacterial strains

To better characterize the MAbs and to determine both the binding capability of the MAbs versus *S*. Abortusequi from different farms and the extent of any cross-reactions against other *Salmonella* serotypes, four *Salmonella* serotypes were used in this study. The five different *S*. Abortusequi strains used in this study were isolated by our laboratory from the tissues of clinically aborted foals. The other three *Salmonella* serotypes, including *S*. Typhimurium, *S*. Enteritidis, and *S*. Dublin, were kind gifts from Prof. Guoqiang Zhu at the College of Veterinary Medicine, Yangzhou University (Yangzhou, Jiangsu, China).

### MAb production and characterization

*S*. Abortusequi recombinant FljB protein was produced as previously described (1). MAbs against FljB were produced through the cell fusion method, with methods that were performed as previously described (35). One Balb/c mouse was primed subcutaneously with purified FljB in Freund complete adjuvant and was boosted intraperitoneally with the same antigen in phosphate-buffered saline (PBS) 3 weeks later. Hybridoma cells were screened using iELISA.

The specificity of the MAbs was further evaluated using iELISA, to compare the pattern of reactivity of the generated MAbs with the *S*. Abortusequi and a spectrum of *Salmonella* of different serotypes. cELISAs were used to analyze the reciprocal competition between the selected MAbs and the natural *S*. Abortusequi infections in horse sera. Direct ELISAs were set up to evaluate the capability of coated MAbs to efficiently bind the HRP-conjugated FljB antigen.

### ELISA for MAb characterization

MAbs were characterized through iELISA, direct ELISA, and cELISA performed as described previously (36). For the iELISAs, five *S*. Abortusequi strains and three other *Salmonella* strains, including *S*. Typhimurium, *S*. Enteritidis, and *S*. Dublin, were adsorbed onto microplates by overnight incubation at 4°C; the plates were then sequentially incubated with hybridoma supernatants and HRP-conjugated goat anti-mouse IgG antibodies for 45 min at 37°C. All washing steps were performed three times with washing buffer (PBS containing 0.1% Tween 20, PBST). After incubation, the plate was washed again and incubated with freshly prepared 3,3′-5,5′-tetramethyl benzidine (TMB) peroxidase substrate (GalaxyBio, Beijing, China) for 10 min at 37°C. The reaction was stopped by adding 2-M H_2_SO_4_, and the optical density at the 450-nm wavelength (OD_450_) was measured using the VersaMax Microplate Reader (BioTek, Winooski, VT, USA).

For the competitive binding assays, cELISAs were designed to analyze the capability of sera naturally infected with *S*. Abortusequi to inhibit the binding of MAbs to *S*. Abortusequi FljB protein. One hundred-microliter samples of *S*. Abortusequi sera were incubated for 45 min at 37°C in FljB antigen-coated plates, and, after washing, 50 µL of hybridoma supernatant was added to each sample. Binding of the MAb was assessed with HRP-conjugated anti-mouse IgG, and the colorimetric reaction was performed as previously described. The competitively reactive MAbs were further evaluated. Briefly, microplates were coated overnight at 4°C with different MAbs at 2-µg/mL concentration, followed by the addition of a serially diluted HRP-conjugated FljB (1:3,000 to 1:9,000).

### cELISA for the detection of *S*. Abortusequi antibody

One hundred microliters of the mixture of the diluted HRP-conjugated FljB antigen with the serially diluted serum sample was incubated for 30 min at 37°C in MAb-coated microplates. After washing three times with PBST, 100 µL of TMB peroxidase substrate (GalaxyBio, Beijing, China) was added to each well, and the plates were incubated for 10 min at 37°C. The absorbance values obtained were expressed as percent inhibition (PI) of the negative control computed as follows: (ODNC – ODSample)/(ODNC – ODPC) × 100%.

In order to standardize the cELISA, the factors that could affect the performance of the assay were assessed by testing a series of sera. The series consisted of one high titer (TAT titer, 32) and one low titer (TAT titer, 2) *S*. Abortusequi-positive serum samples and three other serum samples positive for other *Salmonella* serotypes (*S*. Typhimurium-, *S*. Enteritidis-, and *S*. Dublin-positive sera). Two factors were assessed: (i) reaction strategies of cELISAs and (ii) screening for serum dilution. Two response strategies were assessed. In the first strategy, the mixture of HRP-conjugated FljB antigen with the serially diluted serum sample was first incubated for 30 min at 37°C, and after incubation, the mixture was transferred into the coated antibodies plate and incubated for another 30 min at 37°C. In the second response strategy, the mixture of HRP-conjugated FljB antigen and serially diluted serum sample was incubated directly with the coated antibodies for 30 min at 37°C.

### TAT

To further evaluate and compare the sensitivities of cELISA and TAT, the same serum samples were tested using both cELISA and TAT. TAT was performed as described previously (37). The mixture of the *S*. Abortusequi antigen with the serially diluted serum sample was incubated for 20 hr at 37°C in tubes.

### Statistical analysis

Receiver operator characteristic (ROC) curve analysis was constructed in GraphPad Prism8 (GraphPad Software, USA), which is a statistical tool for evaluating the discriminatory power of a diagnostic test. The area under the ROC curve (AUC) was employed to assess the accuracy of the tests distinguished between non-informative (AUC = 0.5), less accurate (0.5 < AUC ≤ 0.7), moderately accurate (0.7 < AUC ≤ 0.9), highly accurate (0.9 < AUC < 1), and perfect tests (AUC = 1) (38). The Wilson-Brown test was used to determine the 95% confidence intervals (CIs).

## RESULTS

### Selection and characterization of MAbs

In order to evaluate the specificity of the MAbs, hybridomas were screened using parallel iELISA against *S*. Abortusequi as well as *Salmonella* of different types. Only MAbs recognizing the *S*. Abortusequi strain were preserved. The specificity of anti-FljB MAbs was further confirmed using the results of competitive binding assays, and the binding of the various anti-FljB MAbs was inhibited only by *S*. Abortusequi positive sera. This reactivity means that the selected MAbs were suitable for the development of a cELISA.

Based on the results of the iELISAs, eight MAbs specific to *S*. Abortusequi were also tested using cELISAs. Three competitively reactive MAbs (1A10, 3B7, and 3B6) were selected with the cELISAs.

**Fig 1:**
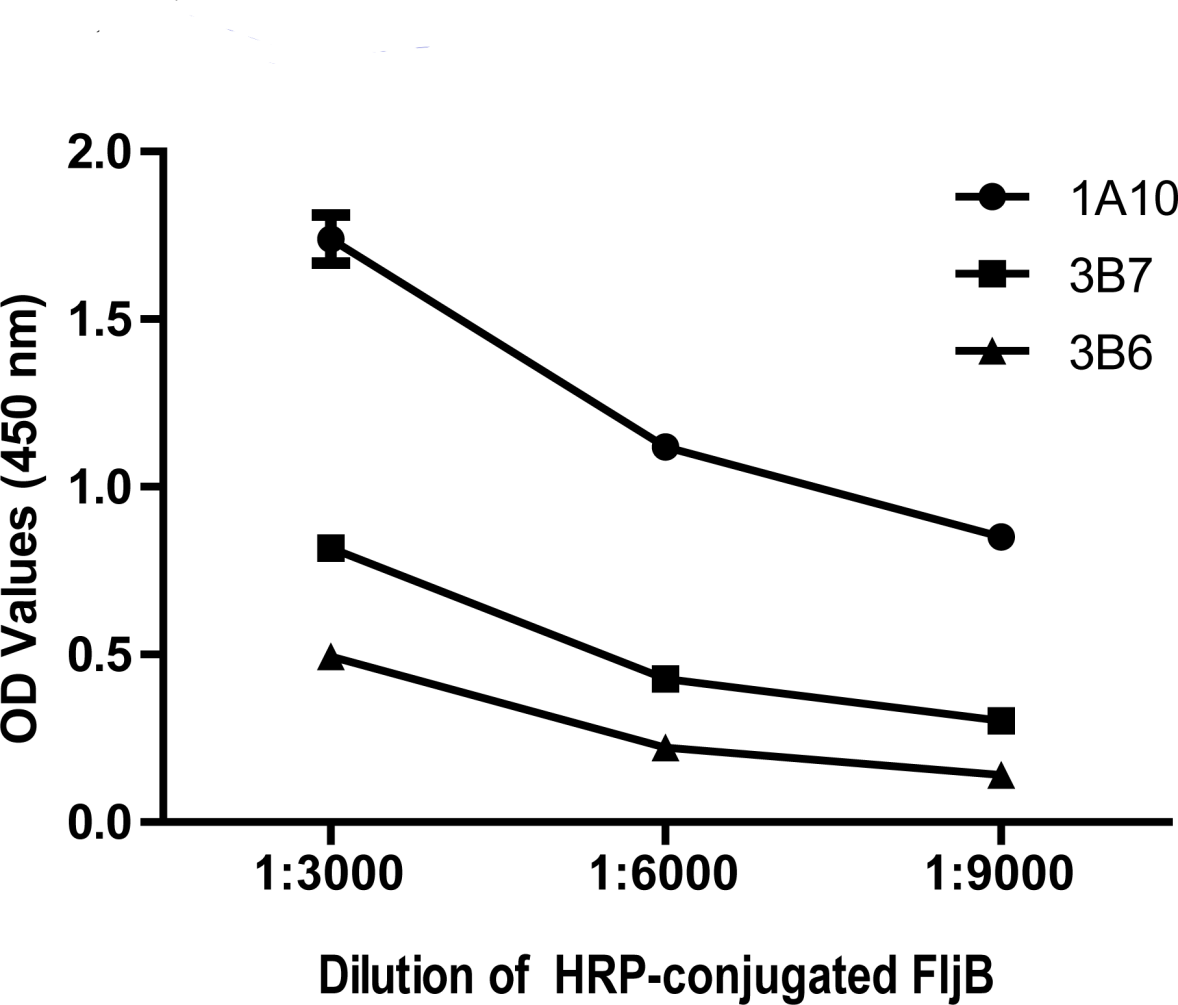
ELISA block titration with the indicated different MAbs and dilutions of HRP-conjugated FljB.

Moreover, the capability of the anti-FljB MAbs to efficiently bind the HRP-conjugated FljB antigen when used as coating antibodies in a direct ELISA was analyzed. Only one coated MAb (1A10) gave the strongest signal and was selected (Fig. 1).

### Setup and standardization of cELISA for the detection of *S*. Abortusequi antibodies

**Fig 2:**
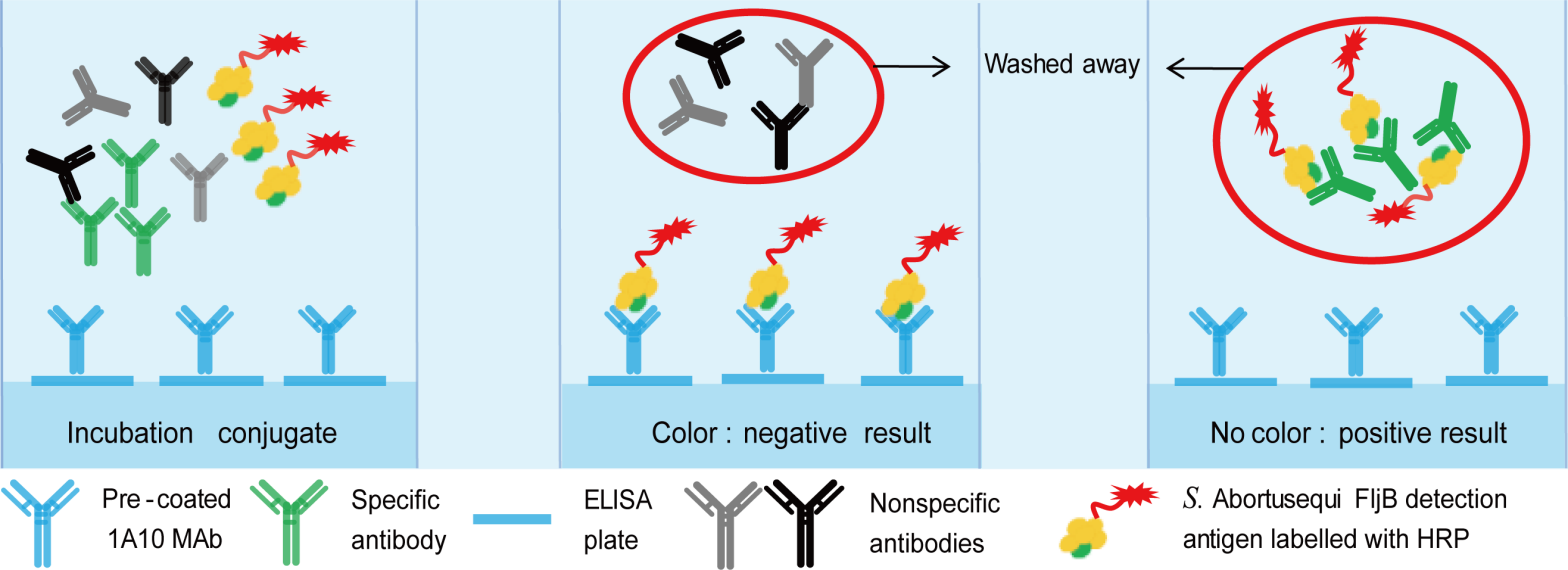
Schematic diagram of solid phase competition ELISA. Anti-*S*. Abortusequi serum and 1A10 MAb, which precoated in the plate, competitively react specifically with FljB-HRP. The conjugation is washed away during the cELISA procedure. Little or no color is visible in the positive samples. Conversely, negative samples have a darker color.

**Fig 3:**
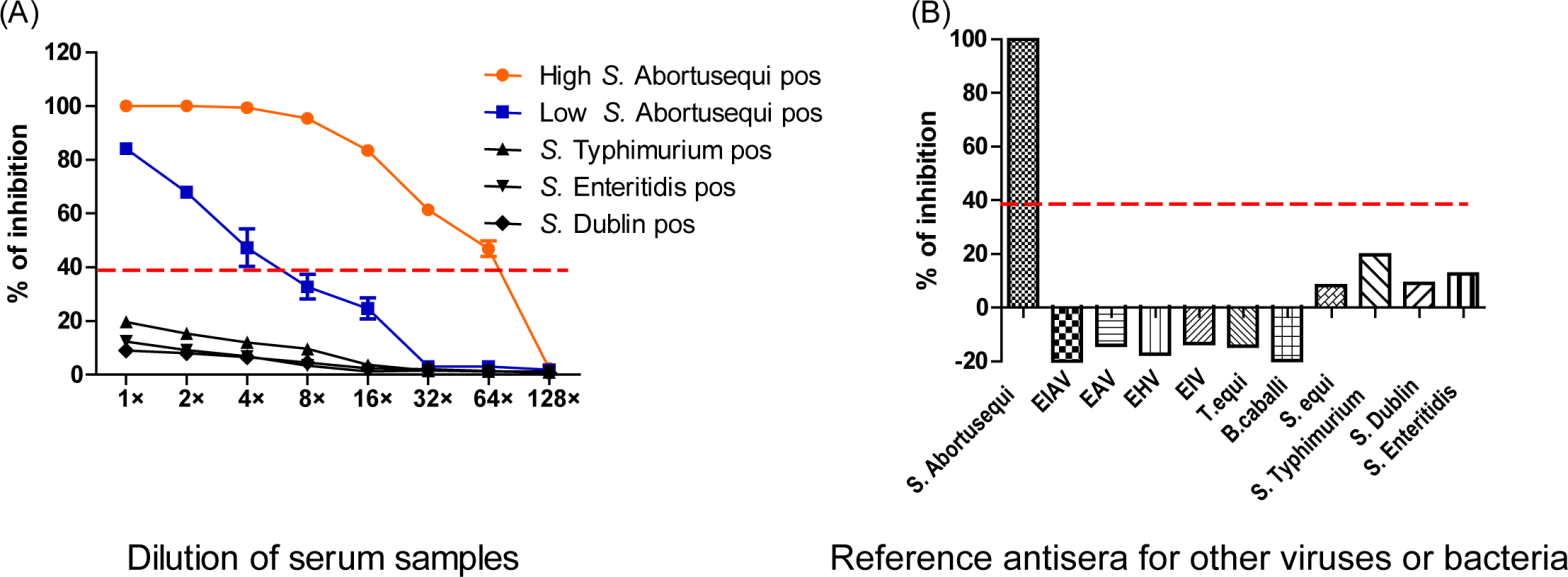
Analytical sensitivity and specificity of the cELISA. (**A**) Titration of strongly and weakly *S*. Abortusequi-positive sera and other three *Salmonella* sera (including sera strongly positive for *S*. Typhimurium, *S*. Enteritidis, and *S*. Dublin) in cELISA. (**B**) Test sera included a standard positive serum of *S*. Abortusequi and reference antisera positive against various pathogens. The dashed line indicates the cutoff value of 39.5% of inhibition.

A cELISA with purified 1A10 MAb adsorbed to the plate was then conducted. The one-step response strategy showed the best performance and speed and was selected for further evaluation (Fig. 2). Then, to confirm the optimal dilution of the test serum, serial dilutions of the five sera, ranging from 1 to 128 times dilutions, were tested with cELISA. A clear-cut separation between *S*. Abortusequi and other three *Salmonella-*positive sera was seen in the cELISA even if the serum was not diluted (Fig. 3A). The maximum dilution that still resulted in a positive value in the cELISA was a dilution of 64 and 4 for high-titer (TAT titer, 32) and low titer (TAT titer, 2) *S*. Abortusequi-positive sera, respectively. The specificity of the detection by the cELISA was investigated using a panel of antisera for other viruses or bacteria. The results showed that for all sera except *S*. Abortusequi-positive serum, no positive results were detected (Fig. 3B). The above results were suggestive of a satisfactory analytical specificity and sensitivity of the cELISA.

### Clinical detection performances

**Fig 4:**
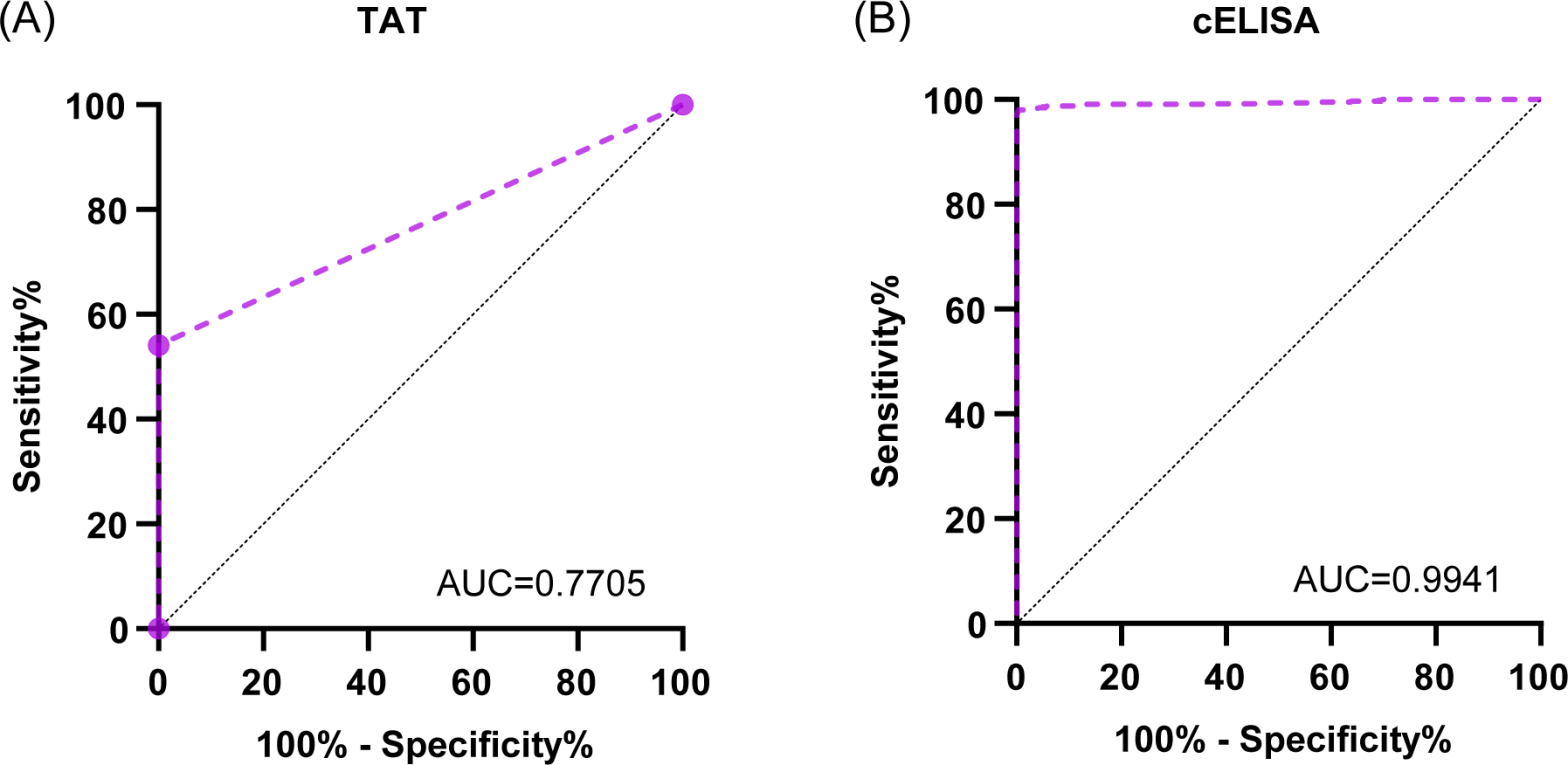
ROC curve analysis. ROC curves for TAT (**A**) and cELISA (**B**) using the panel of 1,175 sera described above.

**Fig 5:**
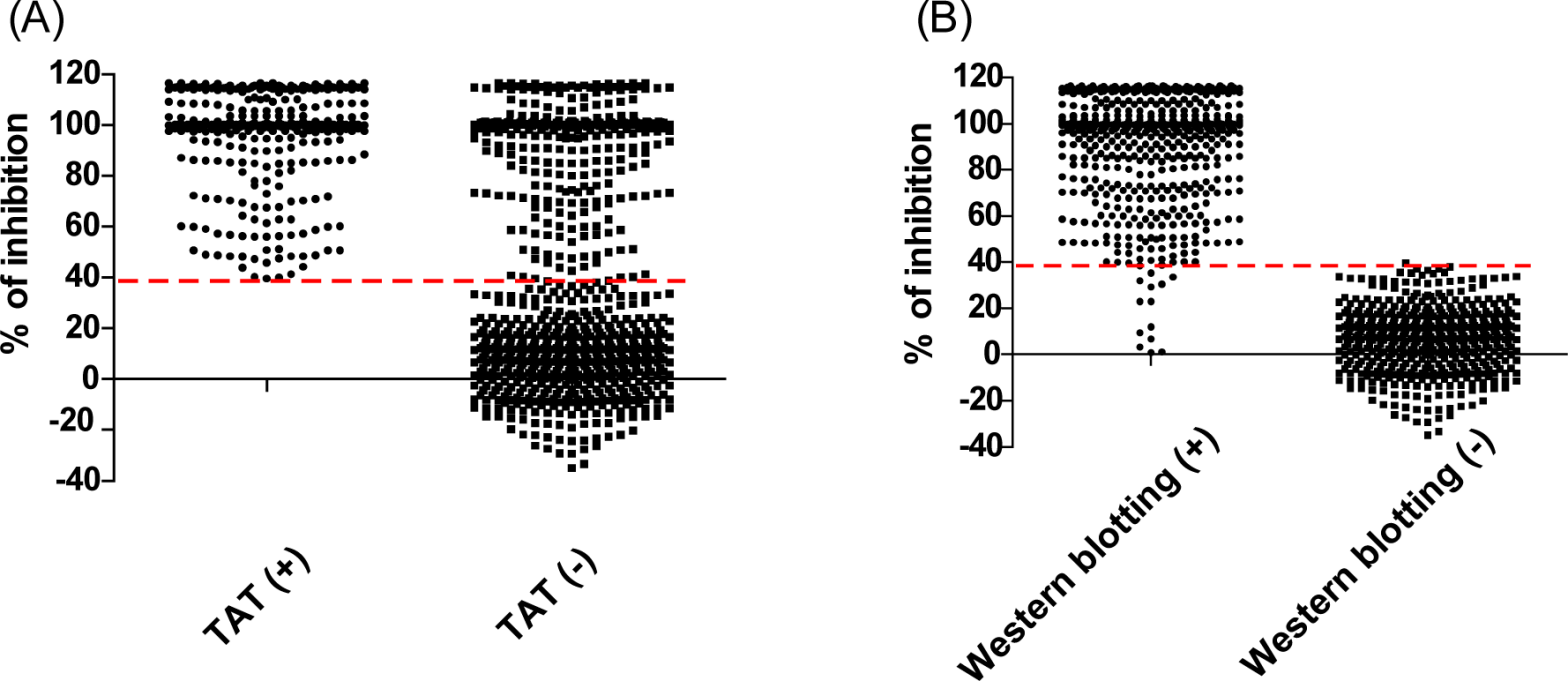
Statistical cELISA inhibition rate distribution based on the TAT (**A**) and Western blotting (**B**) results of serum samples. The dashed line indicates the cutoff value of 39.5% of inhibition.

The assay performances of the cELISA were validated by assessing 1,175 *S*. Abortusequi-positive and *S*. Abortusequi-negative serum samples originating from horses and donkeys (Table 1). Samples were taken from the field and reference sera, were classified as *S*. Abortusequi positive or negative according to their known origin, and were confirmed with Western blotting. The ROC curves were constructed based on the previous classification of the sera into positive and negative. When compared with the Western blotting results, the AUC values showed that the TAT is moderately accurate (AUC = 0.7705; 95% Cl, 0.7437–0.7972) but has very low sensitivity (Se: 54.05; 95% CI, 50.28–57.86) (Fig. 4A; Table 1). Unexpectedly, the AUC values showed that the cELISA (cutoff value: 38.2% of inhibition) is highly accurate (AUC = 0.9941; 95% Cl 0.9898–0.9984) and has very high sensitivity (Se: 98.03; 95% CI, 96.66–98.85) and specificity (Sp: 99.81; 95% CI, 98.91–99.99) (Fig. 4B). Considering the excellent clinical test performance and to avoid false-positive results in routine activity, a cELISA cutoff value of 39.5% was selected with Se and Sp values of 97.58 (95% Cl, 96.10–98.50) and 100 (95% Cl, 99.26–100.00). When the cutoff value of cELISA was determined to be 39.5%, 883 of 1,175 samples tested in the TAT had the same results as the cELISA but 292 had divergent results between the two tests (Fig. 5A), whereas 1,162 of 1,175 samples yielded the same test results in both the Western blotting and cELISA with only 13 samples yielding differing results using the two methods (Fig. 5B).

## DISCUSSION

The re-emergence of *S*. Abortusequi infection in equids has caused serious economic losses to China. Furthermore, horses and donkeys have been found infected with the bacterium in more and more provinces in China (24, 25). *S*. Abortusequi infection could therefore have wider economic impacts than currently predicted.

The major goal of this study was to develop a rapid and simple cELISA method based on a MAb to detect antibodies against *S*. Abortusequi in different animal species. The cELISA described here has a direct advantage over iELISA because secondary antibodies specific to the immunoglobulins of the species being tested are not required. It was developed by using a MAb specific to *S*. Abortusequi and exhibited no cross-reactions to other *Salmonella* strains. We tested two different reaction strategies to select our cELISA and chose that characterized by optimum performance combined with a reduction in assay time compared with the current best available TAT test (Fig. 2). This assay consisted of purified MAb adsorbed to the plate with the addition of the mixtures of HRP-conjugated FljB antigen together with the serum sample in a single step. Interestingly, even when the serum being tested was undiluted, the assay was still able to discriminate well between *S*. Abortusequi-positive and *S*. Abortusequi-negative samples (Fig. 3A).

The developed cELISA was further validated with a large number of serum samples from different species obtained from the field and with other sera against EHV, EIV, EAV, EIAV, *S. equi*, *T. equi*, and *B. caballi* and three *Salmonella* serovars including *S*. Dublin, *S*. Typhimurium, and *S*. Enteritidis (Table 1). Although *Salmonella* is categorized into more than 2,600 *Salmonella* serotypes, there are few serotypes of *Salmonella* causing equine infections that have been reported. *S*. Abortusequi is the most common serotype of *Salmonella* that infects equines in China. According to our data, all *Salmonella* isolates in samples from equine abortions were identified as *S*. Abortusequi serotype. However, *S*. Typhimurium, *S*. Enteritidis, and *S*. Dublin serovars may also cause infection in equines in rare cases (11). Therefore, *S*. Typhimurium, *S*. Enteritidis, and *S*. Dublin serovars were selected to evaluate the specificity of the cELISA. Sera originating from horses strongly positive against other viruses or bacteria were classified as a negative population, better confirming the specificity of the cELISA (Fig. 3B).

The total positive detection rates of the cELISA and TAT in positive serum samples were 98.03% (647/660) and 53.79% (355/660), respectively. The 355 samples that tested positive in the TAT all also tested positive in the cELISA (Fig. 5A). These results showed that the cELISA has excellent detection performance and discriminating power with a high Se and Sp. Moreover, the performance of the cELISA showed a very good agreement (98.89%, 1,162/1,175) with Western blotting (Fig. 5B) compared to the TAT assays (74.21%, 872/1,175)).

In general, the cELISA was in very good agreement with the Western blotting assays, drawing attention to some outliers in this study (Fig. 5B). Thirteen samples originating from horse or donkey farms tested positive by Western blotting but resulted negative in the cELISA. Further analysis showed that the 13 sera were only weakly positive in Western blotting. Considering that these 13 samples originated from areas where *S*. Abortusequi is known to be present, we think that these were not false positives, and we suppose that the lack of correspondence between the two assays could be due to a higher sensitivity of the Western blotting method than cELISA.

Furthermore, a series of other advantageous properties of the cELISA, such as the feasibility of using the sera without dilution, and the one-step response strategy make the cELISA ideal for screening and confirming of *S*. Abortusequi infection in equids. Since positive antibody presumably takes days to develop following infection, this limitation should be noted when the cELISA is used to detect serum samples.

## Data Availability

The data used to support the findings of this study are included within the article.

## References

[1] Mirhosseini SA , Fooladi AAI , Amani J , Sedighian H . 2017. Production of recombinant flagellin to develop ELISA-based detection of Salmonella Enteritidis. Braz J Microbiol 48:774–781. doi:10.1016/j.bjm.2016.04.033 28739413 PMC5628325

[2] Yang Y , Zhang J , Zhu C , Meng X , Sun S , Zhu G . 2019. A promising detection candidate for flagellated Salmonella spp. AMB Express 9:128. doi:10.1186/s13568-019-0851-0 31414324 PMC6694378

[3] Shi C , Singh P , Ranieri ML , Wiedmann M , Moreno Switt AI . 2015. Molecular methods for serovar determination of Salmonella. Crit Rev Microbiol 41:309–325. doi:10.3109/1040841X.2013.837862 24228625

[4] Song JH , Cho H , Park MY , Na DS , Moon HB , Pai CH . 1993. Detection of Salmonella Typhi in the blood of patients with typhoid fever by polymerase chain reaction. J Clin Microbiol 31:1439–1443. doi:10.1128/jcm.31.6.1439-1443.1993 8314983 PMC265558

[5] Sonne-Hansen J , Jenabian SM . 2005. Molecular serotyping of Salmonella: identification of the phase 1 H antigen based on partial sequencing of the fliC gene. APMIS 113:340–348. doi:10.1111/j.1600-0463.2005.apm_113505.x 16011660

[6] Itoh Y , Hirose K , Miyake M , Khan AQ , Hashimoto Y , Ezaki T . 1997. Amplification of rfbE and fliC genes by polymerase chain reaction for identification and detection of Salmonella serovar Enteritidis, Dublin and Gallinarum-Pullorum. Microbiol Immunol 41:791–794. doi:10.1111/j.1348-0421.1997.tb01928.x 9403503

[7] Kumar S , Balakrishna K , Batra HV . 2006. Detection of Salmonella enterica serovar Typhi (S. Typhi) by selective amplification of invA, viaB, fliC-d and prt genes by polymerase chain reaction in mutiplex format. Lett Appl Microbiol 42:149–154. doi:10.1111/j.1472-765X.2005.01813.x 16441380

[8] El-Sayed A , El-Shishtawy M , El-Taweel F , El-Mansoury H . 2015. Multiplex PCR for diagnosis of Salmonella enterica serovar Typhi. Clin. Lab 61:1537–1543. doi:10.7754/Clin.Lab.2015.150115 26642717

[9] Massi MN , Shirakawa T , Gotoh A , Bishnu A , Hatta M , Kawabata M . 2003. Rapid diagnosis of typhoid fever by PCR assay using one pair of primers from flagellin gene of Salmonella Typhi. J Infect Chemother 9:233–237. doi:10.1007/s10156-003-0256-4 14513391

[10] Akiba M , Uchida I , Nishimori K , Tanaka K , Anzai T , Kuwamoto Y , Wada R , Ohya T , Ito H . 2003. Comparison of Salmonella enterica serovar Abortusequi isolates of equine origin by pulsed-field GEL electrophoresis and fluorescent amplified-fragment length polymorphism fingerprinting. Vet Microbiol 92:379–388. doi:10.1016/s0378-1135(02)00422-4 12554106

[11] Traub-Dargatz JL , Garber LP , Fedorka-Cray PJ , Ladely S , Ferris KE . 2000. Fecal shedding of Salmonella spp by horses in the United States during 1998 and 1999 and detection of Salmonella spp in grain and concentrate sources on equine operations. J Am Vet Med Assoc 217:226–230. doi:10.2460/javma.2000.217.226 10909464

[12] Marenzoni ML , Lepri E , Casagrande Proietti P , Bietta A , Coletti M , Timoney PJ , Passamonti F . 2012. Causes of equine abortion, stillbirth and neonatal death in central Italy. Vet Rec 170:1021–1026. doi:10.1136/vr.100551 22368162

[13] Grandolfo E , Parisi A , Ricci A , Lorusso E , Siena R , Trotta A , Buonavoglia D , Martella V , Corrente M . 2018. High mortality in foals associated with Salmonella enterica subsp. enterica abortusequi infection in Italy. J Vet Diagn Invest 30:483–485. doi:https:// 10.1177/1040638717753965 29322884 10.1177/1040638717753965 PMC6505811

[14] Madić J , Hajsig D , Sostarić B , Curić S , Seol B , Naglić T , Cvetnić Z . 1997. An outbreak of abortion in mares associated with Salmonella Abortusequi infection. Equine Vet J 29:230–233. doi:10.1111/j.2042-3306.1997.tb01674.x 9234017

[15] Stritof Z , Habus J , Grizelj J , Koskovic Z , Barbic LJ , Stevanovic V , Tomic DH , Milas Z , Perharic M , Staresina V , Turk N . 2016. Two outbreaks of Salmonella Abortusequi abortion in mares in Croatia. J Equine Vet Sci 39:S63. doi:10.1016/j.jevs.2016.02.135

[16] Buigues S , Ivanissevich A , Vissani MA , Viglierchio V , Minatel L , Crespo F , Herrera M , Timoney P , Barrandeguy ME . 2012. Outbreak of Salmonella abortus equi abortion in embryo recipient polo mares. J Equine Vet Sci 32:S69–S70. doi:10.1016/j.jevs.2012.08.150

[17] Di Gennaro EE , Guida N , Franco PG , Moras EV , Muñoz AJ . 2012. Infectious abortion caused by Salmonella enterica subsp enterica serovar Abortusequi in Argentina. J Equine Vet Sci 32:S74. doi:10.1016/j.jevs.2012.08.158

[18] Neustroev MP , Petrova SG . 2020. Developmental results of a vaccine against Salmonella-induced equine abortion. Russ Agric Sci 46:530–533. doi:10.3103/S1068367420050158 33169058 PMC7641599

[19] Singh BR , Chandra M , Hansda D , Alam J , Babu N , Siddiqui MZ , Agrawal RK , Sharma G . 2013. Evaluation of vaccine candidate potential of deltaaroA, deltahtrA and deltaaroAdeltahtrA mutants of Salmonella enterica subspecies enterica serovar Abortusequi in guinea pigs. Indian J Exp Biol 51:280–287. 24195347

[20] Chandra M , Singh BR , Babu N , Agarwal RK , MZ Siddiqui . 2014. Anti-abortion and fertility vaccine potential of defined double deletion (ΔaroAΔhtrA) mutant (S30) of Salmonella Abortusequi in equids J Equine Vet Sci. 34:765-773.

[21] Hofer E , Berghold C , Sick KJWTM . 2004. Salmonella enterica subsp enterica serovar Abortusequi infections in Equidae in Austria. Wien Tierarztl Monatsschr 91:292–296.

[22] Hong CB , Donahue JM , Giles RC , Petrites-Murphy MB , Poonacha KB , Roberts AW , Smith BJ , Tramontin RR , Tuttle PA , Swerczek TW . 1993. Equine abortion and stillbirth in central Kentucky during 1988 and 1989 foaling seasons. J Vet Diagn Invest 5:560–566. doi:10.1177/104063879300500410 8286455

[23] Niwa H , Hobo S , Kinoshita Y , Muranaka M , Ochi A , Ueno T , Oku K , Hariu K , Katayama Y . 2016. Aneurysm of the cranial mesenteric artery as a site of carriage of Salmonella enterica subsp. enterica serovar Abortusequi in the horse. J Vet Diagn Invest 28:440–444. doi:10.1177/1040638716649640 27271985

[24] Wang J , Guo K , Li S , Liu D , Chu X , Wang Y , Guo W , Du C , Wang X , Hu Z . 2023. Development and application of real-time PCR assay for detection of Salmonella Abortusequi. J Clin Microbiol 61:e0137522. doi:10.1128/jcm.01375-22 36856425 PMC10035326

[25] Wang H , Liu KJ , Sun YH , Cui LY , Meng X , Jiang GM , Zhao FW , Li JJ . 2019. Abortion in donkeys associated with Salmonella abortus equi infection. Equine Vet J 51:756–759. doi:10.1111/evj.13100 30868638

[26] Wang X , Ji Y , Su J , Xue Y , Xi H , Wang Z , Bi L , Zhao R , Zhang H , Yang L , Guo Z , Guan Y , Feng X , Sun C , Lei L , ur Rahman S , Dong J , Han W , Gu J , Julia Pettinari M . 2020. Therapeutic efficacy of phage P IZ SAE-01E2 against abortion caused by Salmonella enterica serovar Abortusequi in mice. Appl Environ Microbiol 86. doi:10.1128/AEM.01366-20 PMC7642090 32887718

[27] Hoelzer K , Moreno Switt AI , Wiedmann M . 2011. Animal contact as a source of human non-typhoidal salmonellosis. Vet Res 42:34. doi:10.1186/1297-9716-42-34 21324103 PMC3052180

[28] Singh IP , Sharma VK , Kaura YK . 1971. Some aspects of the epidemiology of Salmonella abortus-equi infection in equines. Br Vet J 127:378–383. doi:10.1016/s0007-1935(17)37443-2 5106157

[29] WOAH 2022. OIE Terrestrial Manual of Diagnostic Tests and Vaccines for Terrestrial Animals (Terrestrial Manual).

[30] Mizel SB , Bates JT . 2010. Flagellin as an adjuvant: cellular mechanisms and potential. J Immunol 185:5677–5682. doi:10.4049/jimmunol.1002156 21048152 PMC3756556

[31] Rumbo M , Nempont C , Kraehenbuhl JP , Sirard JC . 2006. Mucosal interplay among commensal and pathogenic bacteria: lessons from flagellin and toll-like receptor 5. FEBS Lett 580:2976–2984. doi:10.1016/j.febslet.2006.04.036 16650409

[32] Hiriart Y , Serradell M , Martínez A , Sampaolesi S , Maciel DG , Chabalgoity JA , Yim L , Algorta G , Rumbo M . 2013. Generation and selection of anti-flagellin monoclonal antibodies useful for serotyping Salmonella enterica. Springerplus 2:640. doi:10.1186/2193-1801-2-640 24349948 PMC3862864

[33] Hajam IA , Dar PA , Shahnawaz I , Jaume JC , Lee JH . 2017. Bacterial flagellin—a potent immunomodulatory agent. Exp Mol Med 49:e373. doi:10.1038/emm.2017.172 28860663 PMC5628280

[34] Guo K , Zhang Z , Yang Y , Zhang W , Wang J , Li S , Chu X , Guo W , Liu D , Wang Y , Hu Z , Wang X , Ren L-Z . 2023. Development and application of an iELISA for the detection of antibody against Salmonella Abortusequi. Emerg Infect Dis 2023:1–11. doi:10.1155/2023/1403180 PMC12016898 40303659

[35] Moreno A , Lelli D , Lavazza A , Sozzi E , Zanni I , Chiapponi C , Foni E , Capucci L , Brocchi E . 2019. Mab-based competitive ELISA for the detection of antibodies against influenza D virus. Transbound Emerg Dis 66:268–276. doi:10.1111/tbed.13012 30179314

[36] Moreno A , Brocchi E , Lelli D , Gamba D , Tranquillo M , Cordioli P . 2009. Monoclonal antibody based ELISA tests to detect antibodies against neuraminidase subtypes 1, 2 and 3 of avian influenza viruses in avian sera. Vaccine 27:4967–4974. doi:10.1016/j.vaccine.2009.05.089 19540274

[37] Anzai T , Kamada M , Nakamura M , Yamamoto K , Isayama Y . 1995. Improvement of tube agglutination test for serodiagnosis of equine paratyphoid. J Jpn Vet Med Assoc 48:945–948. doi:10.12935/jvma1951.48.945

[38] Swets JA . 1988. Measuring the accuracy of diagnostic systems. Science 240:1285–1293. doi:10.1126/science.3287615 3287615

